# Correlation Between Chronic Pain Acceptance and Clinical Variables in Ankylosing Spondylitis and Its Prediction Role for Biologics Treatment

**DOI:** 10.3389/fmed.2020.00017

**Published:** 2020-01-31

**Authors:** Ting Li, Yaqun Liu, Rong Sheng, Jian Yin, Xin Wu, Huji Xu

**Affiliations:** ^1^Department of Rheumatology and Immunology, Changzheng Hospital, Second Military Medical University, Shanghai, China; ^2^Beijing Tsinghua Changgung Hospital, School of Clinical Medicine, Tsinghua University, Beijing, China; ^3^Peking-Tsinghua Center for Life Sciences, Tsinghua University, Beijing, China

**Keywords:** ankylosing spondylitis, chronic pain, chronic pain acceptance questionnaire, biologics, anti-TNF treatment, C-reactive protein

## Abstract

**Objectives:** Studies have proven that improving patients' acceptance of chronic pain could be an effective therapy for alleviating pain and other symptoms. Our objectives were to investigate the correlation between chronic pain acceptance and clinical variables in ankylosing spondylitis (AS), and the prediction role of chronic pain acceptance for biologics treatment.

**Methods:** First, 167 AS patients were recruited to complete a series of questionnaires, including the Bath Ankylosing Spondylitis Disease Activity Index (BASDAI), Bath Ankylosing Spondylitis Functional Index (BASFI), Chronic Pain Acceptance Questionnaire (CPAQ), Hospital Anxiety and Depression Scale (HADS), and Tampa Scale for Kinesiophobia (TSK). Bivariate correlation analysis was utilized to investigate the correlation between pain acceptance and clinical variables. Based on the level of chronic pain acceptance and serum C-reactive protein (CRP), patients were separated into four subgroups. Then, another 68 patients initiating anti-tumor necrosis factor (TNF) treatment were recruited to complete the questionnaires at baseline (T0) and 3 months after treatment (T3). The changes in clinical variables and treatment response were compared between multiple subgroups.

**Results:** Chronic pain acceptance had strong correlations with anxiety, depression and fear of movement, and moderate correlations with BASFI and pain intensity. Both activity engagement (AE) and pain willingness (PW) had significant correlations with pain intensity, BASFI and psychological status. In addition, AE had a significant correlation with disease duration, while PW had a significant correlation with ASDAS-CRP. Subgroup analysis showed that patients with low chronic pain acceptance and high levels of serum CRP had the highest BASDAI. Among patients initiating anti-TNF treatment, those with high pain acceptance and high levels of serum CRP achieved the most obvious reduction in BASDAI after 3 months treatment.

**Conclusion:** Pain acceptance is a new tool to assess pain in AS which may also reflect physical and psychological status. Clinicians should identify high-risk patients with low chronic pain acceptance and high levels of serum CRP, and give psychological and pharmacological intervention promptly. Moreover, the combination of baseline chronic pain acceptance and serum CRP level could be used to predict the treatment response in AS patients initiating biologics treatment.

## Introduction

Ankylosing spondylitis (AS) is a chronic inflammatory arthritis that most often affects the sacroiliac joint and spine (1). The major clinical manifestations of AS consist of inflammatory low back pain, morning stiffness, limited spinal mobility, and extra-articular manifestations such as enthesitis and iritis. Pain in AS is usually considered as a surrogate marker for inflammatory, but it is not always parallel with actual inflammatory level (2). Studies have illustrated that AS patients have mechanical and temperature sensation defects on the dorsum their feet, where the skin is innervated by the same spinal nerve as the lower back (3). Additionally, evidence from magnetic resonance imaging suggests that AS patients who are experiencing chronic pain present both functional and structural brain disorder (4). Therefore, pain in AS involves not only inflammatory components but also nociceptive and neuropathic components. This kind of mixed chronic pain could result in depression, anxiety, fear of movement, function limitation and even psychological or physical disability. Based on the complexity of pain in AS, it is not difficult to understand why non-steroidal anti-inflammatory drugs (NSAIDs) and biologics aiming to suppress inflammation do not always reduce pain effectively in many AS patients (1).

In recent years, acceptance and commitment therapy (ACT) aiming to increase patients' acceptance of chronic pain has confirmed to be beneficial to alleviate pain (5, 6). The notion of “pain acceptance” was first introduced by McCracken 20 years ago (7). It refers to one's reaction and adaption to chronic pain (7). This notion potentially encouraged people to focus their attention on living a normal life, participating in meaningful activities, and pursuing their life goals in spite of the presence of chronic pain. Studies have illustrated that patients with higher pain acceptance have more possibility to live with less psychological and physical disability (8, 9). Since then, many studies have demonstrated that positively accepting chronic pain is an effective pain management strategy compared with traditional coping strategies (10, 11). Some studies have suggested that the pain acceptance might be a strong predictor of psychological status and physical function in patients with chronic pain (12–15). Thus, assessing patients' chronic pain acceptance is quite beneficial for clinicians in identifying high-risk individuals and making personalized treatment decisions.

To measure the level of chronic pain acceptance, Geisser et al. developed a 34-item Chronic Pain Acceptance Questionnaire (CPAQ-34) (16). Later, Vowles et al. revised the CPAQ-34 and conducted a 20-item version (CPAQ-20) (17). So far, the CPAQ-20 has been validated with good reliability and validity in many countries, including China (12, 18). However, chronic pain acceptance in AS patients remains unclear.

The objectives of this study were to investigate the correlation between chronic pain acceptance and clinical variables including disease duration, pain intensity, disease activity, activity limitations, depression, anxiety and fear of movement, and to explore the prediction role of chronic pain acceptance for biologics treatment.

## Methods

### Participants

At first, 167 AS patients were recruited for the cross-sectional study. Then, another 68 AS patients initiating anti-tumor necrosis factor (TNF) treatment were recruited to explore the prediction role of chronic pain acceptance for biologics treatment. All the patients were recruited from the Department of Rheumatology and Immunology of Shanghai Changzheng Hospital from April 2016 to September 2017. Patients who had accompanying malignant tumors or other rheumatic diseases and those who were unable to comprehend and answer the questionnaires were excluded. AS was diagnosed by two rheumatologists according to the modified New York criteria (19).

After consenting to this study, participants were given a series of questionnaires to complete. These questionnaires included a form for demographic data, the Bath Ankylosing Spondylitis Disease Activity Index (BASDAI), Bath Ankylosing Spondylitis Functional Index (BASFI), Chronic Pain Acceptance Questionnaire (CPAQ), Hospital Anxiety and Depression Scale (HADS), and Tampa Scale for Kinesiophobia (TSK). The 68 patients initiating anti-TNF treatment were tested at baseline (T0) and 3 months after treatment (T3). All the participants completed the questionnaires independently. This study was approved by the Ethics Committee of Shanghai Changzheng Hospital.

### Outcome Measures

Disease activity of AS was measured by the BASDAI and Ankylosing Spondylitis Disease Activity Score-CRP (ASDAS-CRP). BASDAI consists of six items associated with five major manifestations, including fatigue, axial joint pain, peripheral joint pain, localized tenderness, and morning stiffness (20). ASDAS-CRP was calculated based on BASDAI item 2, 3, and 6, patient's global assessment of diseases activity and CRP (20). Pain intensity was defined as the higher one among item 2 (level of axial joint pain) and item 3 (level of peripheral joint pain) of the BASDAI (20). Patient-reported limitations in physical functions were assessed with the Assessment SpondyloArthritis international Society (ASAS)-endorsed BASFI questionnaire (21). The level of serum CRP served as the laboratory-based inflammatory index and was acquired from patients' medical records. ASAS20 and ASAS40, which measures the percentages of patients who obtain 20 and 40% improvement from baseline to month 3, were used to evaluated the response rate of anti-TNF treatment.

CPAQ-20 was utilized to evaluate the chronic pain acceptance in AS. It is composed of two subscales: pain willingness (PW) and activity engagement (AE) (7, 8). The PW subscale refers to the extent to which a patient trusts that trying to control the feeling of chronic pain is a necessary strategy for them to live a better life. The AE subscale refers to the extent to which a patient participates in everyday activities in spite of the presence of chronic pain. All 20 items are rated on a 6-point numerical rating scale (0 = never true, 6 = always true). The total score was calculated by summing up AE and PW, producing a score between 0 and 120. Higher scores indicate better acceptance. Vowles et al. found that the average CPAQ-20 score of chronic pain patients with low acceptance is 29.1 (Standard deviation = 10.8) (17). Thus, standard deviation (SD) multiplied by 1.64 and then plus the average score, which is 46.8, could be used as the cut-off point to distinguish low and high acceptance. The psychometric properties of the CPAQ-20 were well-established (11, 18). The beliefs of fear of movement were measured by the TSK questionnaire which consists of 17 items. Each item is rated from 1 (strongly disagree) to 4 (strongly agree). The total score was calculated by summing up all the items, producing a score between 17 and 68. Higher scores indicate greater fear of movement. The validity and reliability of the TSK were well-established (22). The severity of anxiety and depression were assessed by the HADS. This 14-item questionnaire consisted of two subscales that were used to evaluate anxiety and depression (23). Each subscale included 7 items, and each item was rated from 0 to 3. The total score of each subscale was calculated by summing up all the related items, producing a score ranging between 0 and 21. Higher score indicated more-severe anxiety and (or) depression. The CPAQ, HADS and TSK in both English and Chinese versions are supplied in the Supplementary Material.

Based on the levels of CRP and CPAQ score, all the patients were separated into four subgroups (Low CRP and low CPAQ, low CRP, and high CPAQ, high CRP, and low CPAQ, high CRP and high CPAQ). The cut-off value to define low and high CRP, low and high CPAQ were 10 mg/L and 46.8, respectively.

### Statistical Analysis

The qualitative variables were presented as frequency and percentages. The quantitative variables were presented as mean and SD. Pearson correlation coefficient was utilizing to perform correlation analysis. The Kruskal-Wallis test (for continuous variables) and chi-square test (for categorical variables) were utilized to compare different variables between multiple subgroups. The Mann-Whitney *U*-test was subsequently used for *post-hoc* analyses with a Bonferroni-corrected *p*-value. A *p*-value of <0.05 was considered statistically significant. All data were analyzed using Statistical Package for the Social Science (SPSS) version 25.0 (SPSS, Inc., IBM, Chicago, USA).

## Results

### Descriptive Data

The 167 participants involved in the cross-sectional study consisted of 144 male and 23 females, aged 35.8 (SD = 11.2) years on average. The disease duration averaged 10.4 (SD = 7.9) years. The average body mass index was 23.6 (SD = 10.5) kg/m^2^. Percentages of patients with peripheral arthritis, enthesitis and uveitis were 30.5, 43.5 and 5.3%, respectively. In addition, 59.3% of the patients had morning stiffness. AS patients had a medium level of CPAQ, averaging at 62.4 (SD = 24.7). For clinical variables, CRP averaged at 13.4 (SD = 12.6) mg/L, while the BASDAI, BASFI, and ASDAS-CRP averaged at 3.8 (SD = 1.9), 3.9 (SD = 2.1), and 2.2 (SD = 0.7), respectively. On the HADS, anxiety averaged at 8.5 (SD = 4.6), and depression averaged at 8.4 (SD = 4.2). The TSK averaged at 36.9 (SD = 12.3) (Table 1). The characteristics of the 68 patients initiating anti-TNF treatment were also shown in Table 1.

**Table 1:** Demographic characteristics of patients with ankylosing spondylitis (*n* = 167) and patients initiating anti-TNF treatment (*n* = 68).

**Characteristics**	**Ankylosing spondylitis *n* = 167**	**Patients initiating anti-TNF treatment *n* = 68**
Age, mean (SD)	35.8 (11.2)	28 (9.0)
Male, *n* (%)	144 (86.2%)	62 (91.2%)
Disease duration (years), mean (SD)	10.4 (7.9)	5.5 (4.5)
Body mass index (kg/m^2^), mean (SD)	23.6 (10.5)	23.2 (9.8)
Peripheral arthritis, *n* (%)	51 (30.5%)	14 (20.6%)
Enthesitis, *n* (%)	73 (43.5%)	24 (35.3%)
Uveitis, *n* (%)	9 (5.3%)	3 (4.4%)
Morning stiffness, *n* (%)	99 (59.3%)	62 (91.1%)
CRP (mg/L), mean (SD)	13.4 (12.6)	17.5 (16.7)
BASDAI, mean (SD)	3.8 (1.9)	5.7 (1.8)
BASFI, mean (SD)	3.9 (2.1)	5.2 (1.8)
ASDAS-CRP, mean (SD)	2.2 (0.7)	2.8 (0.7)
CPAQ, mean (SD)	62.4 (24.7)	57.9 (24.3)
AE, mean (SD)	29.2 (13.3)	25.3 (15.1)
PW, mean (SD)	33.2 (12.8)	32.6 (17.5)
HADS		
Anxiety, mean (SD)	8.5 (4.6)	6.8 (4.6)
Depression, mean (SD)	8.4 (4.2)	6.3 (3.9)
TSK, mean (SD)	36.9 (12.3)	42.4 (15.3)

*TNF, tumor necrosis factor; SD, standard deviation; n, number; CRP, C-reactive protein; BASDAI, Bath Ankylosing Spondylitis Disease Activity Index; BASFI, Bath Ankylosing Spondylitis Functional Index; ASDAS, Ankylosing Spondylitis Disease Activity Score; CPAQ, Chronic Pain Acceptance Questionnaire; AE, activity engagement; PW, pain willingness; HADS, Hospital Anxiety and Depression Scale; TSK, Tampa Scale for Kinesiophobia*.

### Correlation Between CPAQ, CPAQ Subscales, and Other Variables

CPAQ had strong correlations with anxiety (*r* = −0.60, *p* < 0.05), depression (*r* = −0.63, *p* < 0.05) and TSK (*r* = −0.69, *p* < 0.01), moderate correlations with BASFI (*r* = −0.54, *p* < 0.05) and pain intensity (*r* = −0.48, *p* < 0.05), and no significant correlations with disease duration (*r* = −0.04, *p* > 0.05), BASDAI (*r* = 0.02, *p* > 0.05), ASDAS-CRP (*r* = −0.14, *p* > 0.05), and CRP (*r* = −0.04, *p* > 0.05). Both AE and PW had significant correlations with pain intensity (*r* = −0.35, *p* < 0.05; *r* = −0.60, *p* < 0.05), BASFI (*r* = −0.48, *p* < 0.05; *r* = −0.32, *p* < 0.05), anxiety (*r* = −0.57, *p* < 0.05; *r* = −0.55, *p* < 0.05), depression (*r* = −0.69, *p* < 0.05; *r* = −0.43, *p* < 0.05), and TSK (*r* = −0.65, *p* < 0.01; *r* = −0.41, *p* < 0.01). In addition, AE had a significant correlation with disease duration (*r* = −0.32, *p* < 0.05), while PW had a significant correlation with ASDAS-CRP (*r* = −0.15, *p* < 0.05) (Table 2).

**Table 2:** Correlations between CPAQ, AE, PW, and clinical variables.

	**CPAQ**	**AE**	**PW**
Disease duration	ns	−0.32^*^	ns
BASDAI	ns	ns	ns
ASDAS-CRP	ns	ns	−0.15^*^
Pain intensity	−0.48^*^	−0.35^*^	−0.60^*^
BASFI	−0.54^*^	−0.48^*^	−0.32^*^
Anxiety	−0.60^*^	−0.57^*^	−0.55^*^
Depression	−0.63^*^	−0.69^*^	−0.43^*^
TSK	−0.69^**^	−0.65^**^	−0.41^**^
CRP	ns	ns	ns

*CPAQ, Chronic Pain Acceptance Questionnaire; AE, activity engagement; PW, pain willingness; BASDAI, Bath Ankylosing Spondylitis Disease Activity Index; BASFI, Bath Ankylosing Spondylitis Functional Index; TSK, Tampa Scale for Kinesiophobia CRP, C-reactive protein; ASDAS, Ankylosing Spondylitis Disease Activity Score; ns, no significant statistical difference*;

* *p <0.05*,

** *p <0.01. P-value was calculated using Pearson correlation test. Data are presented as Pearson correlation coefficient*.

### Characteristics of Groups Based on CPAQ and CRP

**Figure 1:**
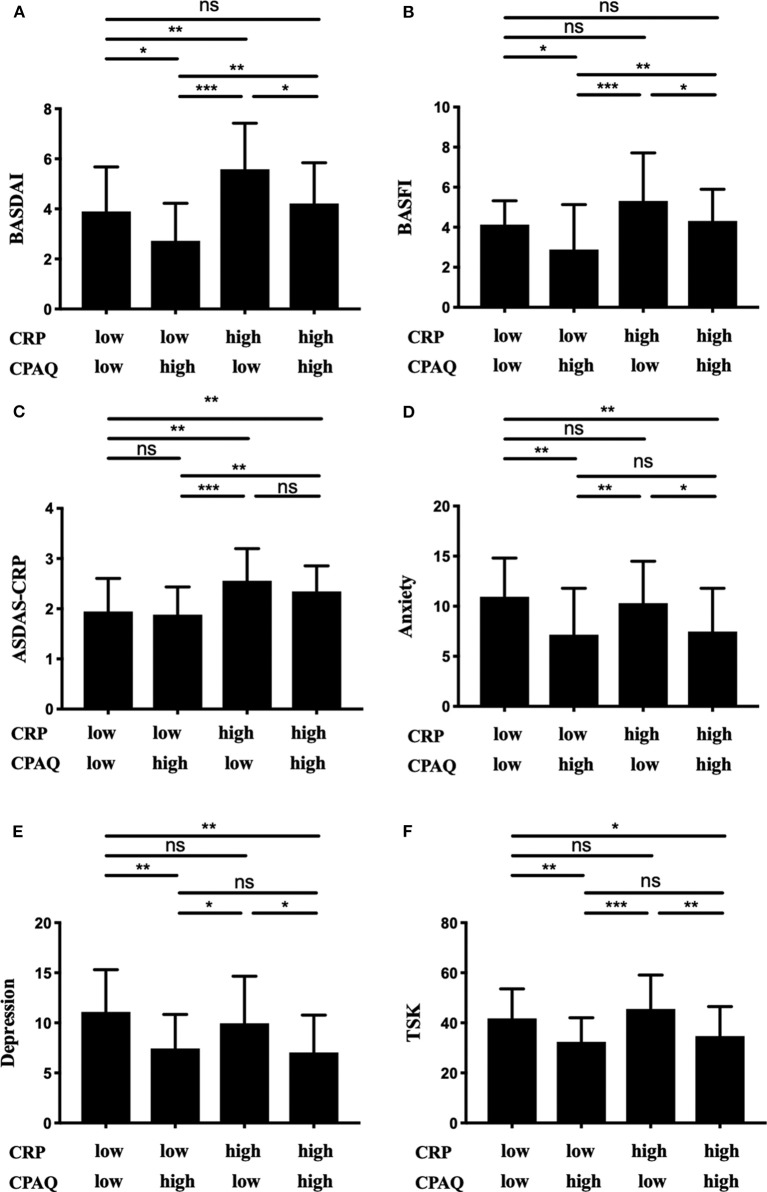
The 167 patients were grouped into four subgroups according to the level of CRP and CPAQ. BASDAI **(A)**, BASFI **(B)**, ASDAS-CRP **(C)**, anxiety **(D)**, depression **(E)**, and TSK **(F)** are shown separately. *p < 0.05; **p < 0.01; ***p < 0.001; ns, no significant difference; CRP, C-reactive protein; CPAQ, Chronic Pain Acceptance Questionnaire; BASDAI, Bath Ankylosing Spondylitis Disease Activity Index; BASFI, Bath Ankylosing Spondylitis Functional Index; ASDAS, Ankylosing Spondylitis Disease Activity Score.

All 167 participants were separated into four groups according to the level of CRP and CPAQ. Subgroup analysis showed that the BASDAI and BASFI were highest in group 3 (low CPAQ and high CRP) and lowest in group 2 (high CPAQ and low CRP). Group 1 (low CPAQ and low CRP) and group 4 (high CPAQ and high CRP) were at the medium level. In addition, group 3 and group 4 had higher ASDAS-CRP than group 1 and group 2, while there were no significant differences between group 3 and group 4 as well as between group 1 and group 2. As for anxiety, depression and TSK, groups with low CPAQ (group 1 and group 3) had higher scores than groups with high CPAQ (group 2 and group 4), while there were no significant differences between group 1 and group 3 or between group 2 and group 4 (Table 3; Figure 1).

**Table 3:** Characteristics in the four groups (*n* = 167).

**Characteristics mean (SD)**	**Group 1 low CRP and low CPAQ, *n* = 31**	**Group 2 low CRP and high CPAQ, *n* = 61**	**Group 3 high CRP and low CPAQ, *n* = 29**	**Group 4 high CRP and high CPAQ, *n* = 46**	***p*^#^**
CRP^a^	4.9 (2.5)	5.4 (2.1)	27.0 (8.8)	21.0 (14.6)	<0.001
CPAQ^b^	37.0 (7.3)	75.6 (16.8)	33.5 (8.3)	80.1 (16.1)	<0.001
AE	16.9 (5.3)	34.9 (10.4)	15.1 (5.9)	38.7 (9.9)	<0.001
PW	20.1 (6.1)	40.7 (8.5)	18.4 (5.2)	41.4 (8.0)	<0.001
BASDAI	3.9 (1.8)	2.7 (1.5)	5.6 (1.8)	4.2 (1.6)	<0.001
BASFI	4.1 (1.2)	2.9 (2.2)	5.3 (2.4)	4.3 (1.6)	<0.001
ASDAS-CRP	2.0 (0.7)	1.9 (0.6)	2.6 (0.6)	2.3 (0.5)	<0.001
Anxiety	10.9 (3.9)	7.1 (4.6)	10.3 (4.2)	7.5 (4.3)	<0.001
Depression	11.1 (4.2)	7.4 (3.4)	10.0 (4.7)	7.0 (3.7)	<0.001
TSK	41.8 (11.8)	32.4 (9.6)	45.6 (13.5)	34.7 (11.8)	<0.001

*SD, standard deviation; n, number; CRP, C-reactive protein; CPAQ, Chronic Pain Acceptance Questionnaire; AE, activity engagement; PW, pain willingness; BASDAI, Bath Ankylosing Spondylitis Disease Activity Index; BASFI, Bath Ankylosing Spondylitis Functional Index; TSK, Tampa Scale for Kinesiophobia. ASDAS, Ankylosing Spondylitis Disease Activity Score*.

# *Kruskal-Wallis test of the four groups*.

a *The cut-off value to define low and high CRP is 10 mg/L*.

b *The cut-off value to define low and high CPAQ is 46.8*.

### Effectiveness of Anti-TNF Treatment in Different Cohorts

The 68 patients initiating anti-TNF treatment were separated into four cohorts according to CPAQ and CRP at baseline as previous described (Table 4). Three months after treatment, ΔCRP, ΔCPAQ, ΔBASDAI, ΔBASFI, and ΔASDAS-CRP were calculated (Table 4). There were significant differences in ΔCRP, ΔBASDAI, and ΔASDAS-CRP among multiple cohorts. Subgroup analysis showed that cohort 4 (high CPAQ and high CRP) had the highest ΔBASDAI, while cohort 1 (low CPAQ and low CRP) had the lowest. ΔBASDAI in cohort 2 and cohort 3 were at the medium level (Table 4; Figure 2A). In addition, cohort 4 had higher ΔASDAS-CRP than cohort 1 and cohort 2, while there were no significant differences in ΔASDAS-CRP between cohort 3 and cohort 4 (Table 4; Figure 2B). There were no significant differences among multiple groups in ΔCPAQ, ΔBASFI, ASAS20, and ASAS40 (Table 4).

**Table 4:** Characteristics at baseline, month 3 and the changes from baseline to month 3 in the four cohorts (*n* = 68).

**Characteristics mean (SD)**		**Cohort 1 low CRP and low CPAQ, *n* = 17**	**Cohort 2 low CRP and high CPAQ, *n* = 15**	**Cohort 3 high CRP and low CPAQ, *n* = 19**	**Cohort 4 high CRP and high CPAQ, *n* = 17**	**p^#^**
CRP	T0	6.1 (2.3)	5.4 (2.1)	26.1 (8.3)	28.0 (12.1)	<0.0001
	T3	5.4 (2.5)	5.2 (2.2)	15.4 (6.5)	17.1 (7.2)	
	Δ	0.7 (0.2)	0.2 (0.1)	10.7 (4.3)	10.9 (4.7)	
CPAQ	T0	40.6 (7.8)	78.0 (16.7)	36.6 (9.2)	81.3 (16.7)	ns
	T3	42.7 (8.5)	76.7 (15.0)	38.0 (7.5)	83.0 (15.0)	
	Δ	−2.1 (0.8)	1.3 (0.5)	−1.4 (0.6)	−2.7 (1.1)	
BASDAI	T0	4.8 (1.4)	4.5 (1.5)	6.8 (1.5)	6.4 (1.4)	<0.0001
	T3	3.8 (1.5)	3.0 (1.2)	4.9 (1.1)	3.9 (1.4)	
	Δ	1.0 (0.4)	1.5 (0.6)	1.8 (0.5)	2.5 (0.4)	
BASFI	T0	4.5 (1.0)	4.2 (1.3)	7.1 (1.7)	6.4 (1.5)	ns
	T3	3.9 (0.8)	3.2 (1.3)	6.0 (1.2)	5.0 (1.0)	
	Δ	0.6 (0.1)	1.0 (0.3)	1.1 (0.4)	1.4 (0.4)	
ASDAS-CRP	T0	2.5 (0.5)	2.2 (0.6)	3.4 (0.7)	3.2 (0.6)	
	T3	1.6 (0.6)	1.3 (0.4)	2.2 (0.7)	1.8 (0.6)	
	Δ	0.9 (0.3)	0.9 (0.3)	1.2 (0.4)	1.4 (0.3)	<0.001
ASAS20	T3	14 (82%)	12 (80%)	16 (84%)	16 (94%)	ns
ASAS40	T3	8 (47%)	8 (53%)	9 (47%)	10 (59%)	ns

*SD, standard deviation; CRP, C-reactive protein; CPAQ, Chronic Pain Acceptance Questionnaire; Δ, changes of scores from baseline (T0) to Month 3 (T3); BASDAI, Bath Ankylosing Spondylitis Disease Activity Index; BASFI, Bath Ankylosing Spondylitis Functional Index; TSK, Tampa Scale for Kinesiophobia; ASDAS, Ankylosing Spondylitis Disease Activity Score; ASAS20, Assessment of SpondyloArthritis International Society 20% improvement response rate; ASAS40, Assessment of SpondyloArthritis International Society 40% improvement response rate*.

# *Kruskal-Wallis test was used to compare ΔCRP, ΔCPAQ, ΔBASDAI, ΔBASFI, ΔASDAS-CRP among the four cohorts. Chi-square test was used compare ASAS20 and ASAS40 among the four cohorts*.

**Figure 2:**
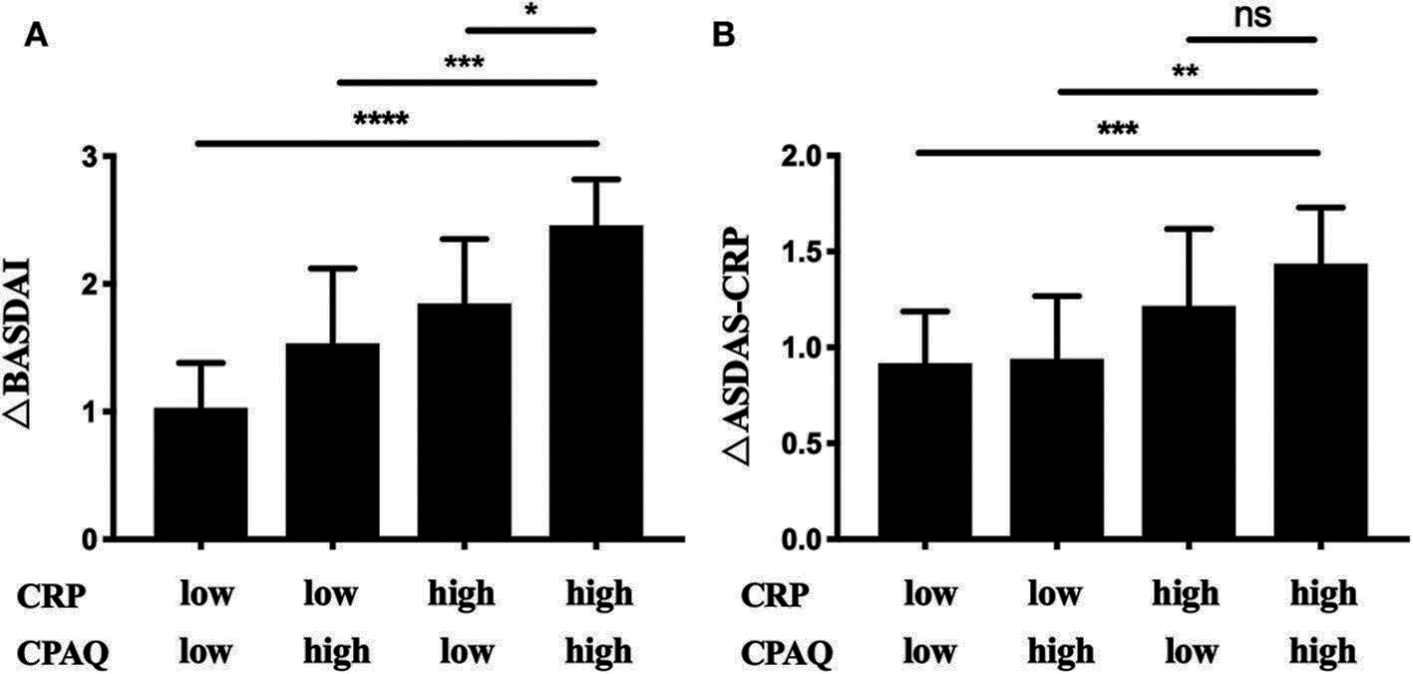
The 68 patients initiating anti-TNF treatment were grouped into 4 subgroups according to the level of CRP and CPAQ. ΔBASDAI **(A)** and ΔASDAS-CRP **(B)** was calculated after 3 months. *p < 0.05, **p < 0.01, ***p < 0.001, ****p < 0.0001. CRP, C-reactive protein; CPAQ, Chronic Pain Acceptance Questionnaire; BASDAI, Bath Ankylosing Spondylitis Disease Activity Index; ASDAS, Ankylosing Spondylitis Disease Activity Score.

## Discussion

To the best of our knowledge, this is the first study to uncover the role of chronic pain acceptance in AS. The mean CPAQ score acquired from all the 167 participants was 62.4 (SD = 24.7), which was lower than that of patients with rheumatoid arthritis, as reported by Ahlstrand et al. (24). Thus, these findings suggest that there exist complex interactions between different diseases and patients' psychological properties.

Correlation analysis revealed that chronic pain acceptance was inversely associated with pain intensity and BASFI rather than with disease duration, BASDAI, ASDAS-CRP, and CRP, indicating that CPAQ might remain stable in the long course of disease without psychological intervention, and was related with pain evaluation and physical functions of AS patients. Indeed, the pathogenesis of pain in AS is not only associated with inflammation but also with psychological factors such as pain acceptance in a great extent. Pain acceptance, as a psychological property, also tended to exert long-term effects on both the psychological and physical status of patients with AS. Thus, pain acceptance is a new tool to assess pain in AS. Without psychological intervention, pain acceptance could be regarded as a long-term stable psychological property and might not be affected by short-term fluctuations of disease. CPAQ is composed of two subscales which are AE and PW. AE had a significant correlation with disease duration and higher correlation with BASFI, indicating that patients with longer disease duration and poor physical functions were less likely to take part in everyday activities. PW had a significant correlation with ASDAS-CRP and higher correlation with pain intensity, indicating that higher willingness to control the feeling of chronic pain may lead to lower pain intensity and ASDAS-CRP. Therefore, it is necessary to advise AS patients with lower AE to participate more in proper exercise. For those with lower PW, clinicians should educate them with effective chronic pain management skills.

Correlations were also found between pain acceptance and psychological status, including depression, anxiety, and fear of movement. Lethem et al. suggested a fear-avoidance model in which avoidant behaviors of patients with chronic pain were more likely to result in anxiety or depression, while these negative emotions would further reinforce patients' fear of movement (25). To a great extent, fear of movement could also be regarded as low level of chronic pain acceptance. Thus, it was not difficult to understand that TSK, the well-established questionnaire used to assess fear of movement, had a strong inverse correlation with CPAQ. Compared with anxiety, depression had a higher correlation with the CPAQ score. This result was consistent with previous reports and suggests that patients with lower levels of chronic pain acceptance are more likely to get into depression. Thus, clinicians should focus much on the psychological status of patients with lower levels of chronic pain acceptance. Proper psychological interventions should be given if necessary.

Based on the level of chronic pain acceptance and serum CRP, patients could be separated into four subgroups. We found that the patients with low chronic pain acceptance and high CRP levels had significantly higher BASDAI than the other groups. Thus, for clinicians, it is necessary to identify patients in this group. And psychological and pharmacological interventions are urgently needed to achieve both psychological and physical improvement.

For the 68 patients initiating anti-TNF treatment, CPAQ did not change significantly after a 3 month anti-TNF treatment. These results further suggested that the pain acceptance of patients is stable and may not change in a short period without psychological inventions. Subgroup analysis indicated that the patients with high pain acceptance and high levels of serum CRP at baseline obtained the highest ΔBASDAI, while the patients with low pain acceptance and low levels of serum CRP at baseline obtained the lowest ΔBASDAI. Until now, there have been no well-established tools to predict the effectiveness of biologics treatment. Although some studies have demonstrated that AS patients with a higher level of CRP at the baseline would acquire a larger improvement on the BASDAI when initiating biologics treatment (26, 27), it is non-negligible that both physical and psychological factors could affect treatment effectiveness and CRP could be affected by many factors, such as age and infection. Our study emphasized the well-predictable role of chronic pain acceptance in combination with serum CRP, and provides new insight into patient stratification.

This study has several limitations. First, magnetic resonance imaging (MRI) was not included to define disease activity and inflammation more accurately. To date, there is no gold standard for the assessment of disease activity in AS. Sometimes CRP and BASDAI may not reflect the actual disease activity and inflammation, and MRI is mostly used for diagnostic purposes (20). Secondly, participants in our cross-sectional study had received different treatments. Further research is needed to investigate the chronic pain acceptance of treatment-naïve patients with AS. Thirdly, in the 68 patients initiating anti-TNF treatment, we investigated the change of clinical variables only 3 months after anti-TNF treatment. Thus, it is unclear whether these changes would occur over a longer period. Finally, our study is a cross sectional study only and further longitudinal study with large sample size is needed to explore more clinical significance and application.

Taken together, we found that lower chronic pain acceptance was correlated with higher pain intensity, higher BASFI and worse psychological status in AS. Pain acceptance is a new tool to assess pain in AS which may also reflect physical and psychological status. Patients with lower levels of chronic pain acceptance and higher levels of serum CRP are more likely to have higher BASDAI. Thus, it is necessary for clinicians to identify these individuals and provide psychological and pharmacological interventions promptly. Moreover, the combination of baseline chronic pain acceptance and serum CRP level could be used to predict the treatment response in AS patients initiating biologics treatment.

## Data Availability Statement

The datasets generated for this study are available on request to the corresponding author.

## Ethics Statement

The studies involving human participants were reviewed and approved by Shanghai Changzheng hospital. The patients/participants provided their written informed consent to participate in this study.

## Author Contributions

HX, TL, and YL conceived and conducted the study. TL and YL analyzed, interpreted the data, and participated in drafting manuscript. HX, TL, and YL revised the manuscript. XW, RS, and JY made substantial contributions to the participant recruitment and data collection. All the authors read and approved the final manuscript.

### Conflict of Interest

The authors declare that the research was conducted in the absence of any commercial or financial relationships that could be construed as a potential conflict of interest.
