# Supplementary material for: Correlation Between Chronic Pain Acceptance and Clinical Variables in Ankylosing Spondylitis and Its Prediction Role for Biologics Treatment
Source: Front Med (Lausanne). 2020 Jan 31;7:17. doi: 10.3389/fmed.2020.00017 (PMC7005047; doi:10.3389/fmed.2020.00017)
Supplement: Supplementary file 1 [file Data_Sheet_1.PDF]

你接受自己的慢性疼痛吗？请完成下面 20 道问题，每个问题有 0-6 个答案，请在自己觉得最适合的下面划√

	绝对不是这样的	极少情况是这样的	偶尔是这样的	有时候是这样的	经常是这样的	通常情况是这样的	完全是这样的
1. 无论有多痛，我都会继续生活。	0	1	2	3	4	5	6
2. 尽管有慢性疼痛，我也会生活很好。	0	1	2	3	4	5	6
3. 我是可以忍受疼痛的。	0	1	2	3	4	5	6
4. 为了减轻疼痛，我愿意牺牲生活中一些重要的事情	0	1	2	3	4	5	6
5. 对我来说，即使不控制疼痛，也能过得很好。	0	1	2	3	4	5	6
6. 世事变迁，尽管有慢性疼痛，我依然过着正常的生活。	0	1	2	3	4	5	6
7. 我需要集中精力以去除疼痛。	0	1	2	3	4	5	6
8. 我做很多事情时会感到疼痛。	0	1	2	3	4	5	6
9. 虽然我有慢性疼痛，但我觉得我的生活很圆满。	0	1	2	3	4	5	6
10. 相比其他生活目标，缓解疼痛并不重要。	0	1	2	3	4	5	6

	绝对不是这样的	极少情况是这样的	偶尔是这样的	有时候是这样的	经常是这样的	通常情况是这样的	完全是这样的
11. 我一定要改变对疼痛的感觉和态度，这样才能在生活中迈出重要一步。	0	1	2	3	4	5	6
12. 尽管有慢性疼痛，我依然坚持我自己生活的轨迹。	0	1	2	3	4	5	6
13. 不论做什么事情，首先要做的都是将疼痛控制在可承受范围内。	0	1	2	3	4	5	6
14. 当我做出重要计划前，首先要控制我的疼痛。	0	1	2	3	4	5	6
15. 即使疼痛加重，我仍然可以履行责任和义务。	0	1	2	3	4	5	6
16. 如果我能控制我对疼痛的负面想法，我可以更好的生活。	0	1	2	3	4	5	6
17. 我会刻意回避加剧疼痛的人和事。	0	1	2	3	4	5	6
18. 我真的对疼痛感到焦虑和恐惧。	0	1	2	3	4	5	6
19. 能够接受疼痛的存在并继续生活，对我来说是一个解脱。	0	1	2	3	4	5	6
20. 当我感受到疼痛时，我不得不比常人付出更多才能将事情做好。	0	1	2	3	4	5	6
