# Supplementary material for: Correlation Between Chronic Pain Acceptance and Clinical Variables in Ankylosing Spondylitis and Its Prediction Role for Biologics Treatment
Source: Front Med (Lausanne). 2020 Jan 31;7:17. doi: 10.3389/fmed.2020.00017 (PMC7005047; doi:10.3389/fmed.2020.00017)
Supplement: Supplementary file 2 [file Data_Sheet_2.PDF]

Chronic Pain Acceptance Questionnaire

Directions: Below you will find a list of statements. Please rate the truth of each statement as it applies to you. Use the following rating scale to make your choices. For instance, if you believe a statement is "always true," you would write a 6 in the blank next to that statement.

0	*1*	*2*	*3*	*4*	*5*	*6*
Never true	Very rarely true	Seldom true	Sometimes true	Often true	Almost always true	Always true

1. I am getting on with the business of living no matter what my level of pain is . . . .
2. My life is going well, even though I have chronic pain . . . . . .
3. It's OK to experience pain . . . . . .
4. I would gladly sacrifice important things in my life to control this pain better . . . . . .
5. It's not necessary for me to control my pain in order to handle my life well . . . . . .
6. Although things have changed, I am living a normal life despite my chronic pain . . . . . .
7. I need to concentrate on getting rid of my pain . . . . . .
8. There are many activities I do when I feel pain . . . . . .
9. I lead a full life even though I have chronic pain . . . . . .
10. Controlling pain is less important than any other goals in my life . . . . . .
11. My thoughts and feelings about pain must change before I can take important steps in my life . . . . . .
12. Despite the pain, I am now sticking to a certain course in my life . . . . . .
13. Keeping my pain level under control takes first priority whenever I'm doing something . . . . . .
14. Before I can make any serious plans, I have to get some control over my pain . . . . . .
15. When my pain increases, I can still take care of my responsibilities . . . . . .
16. I will have better control over my life if I can control my negative thoughts about pain . . . . . .
17. I avoid putting myself in situations where my pain might increase . . . . . .
18. My worries and fears about what pain will do to me are true . . . . . .
19. It's a relief to realize that I don't have to change my pain to get on with my life . . . . . .
20. I have to struggle to do things when I have pain . . . . . .

Scoring

Activities Engagement: Sum items 1, 2, 3, 5, 6, 8, 9, 10, 12, 15, and 19.

Pain Willingness: Reverse score items 4, 7, 11, 13, 14, 16, 17, 18, and 20.

Total score: Sum the scores of the two subscales (Activities Engagement and Pain Willingness).
