# Supplementary material for: Correlation Between Chronic Pain Acceptance and Clinical Variables in Ankylosing Spondylitis and Its Prediction Role for Biologics Treatment
Source: Front Med (Lausanne). 2020 Jan 31;7:17. doi: 10.3389/fmed.2020.00017 (PMC7005047; doi:10.3389/fmed.2020.00017)
Supplement: Supplementary file 3 [file Data_Sheet_3.PDF]

请阅读以下各个项目，在其中最符合你过去一个月的情绪选项上打勾。

1、我感到紧张或痛苦：
☐ 根本没有
☐ 有时候
☐ 大多时候
☐ 几乎所有时候

2、我对以往感兴趣的事情还是有兴趣：
☐ 肯定一样
☐ 不像以前那样多
☐ 只有一点
☐ 基本上没有了

3、我感到有点害怕，好像预感到什么可怕的事情要发生：
☐ 根本没有
☐ 有一点，但并不使我苦恼
☐ 是有，不太严重
☐ 非常肯定和十分严重

4、我能够哈哈大笑，并看到事物好的一面：
☐ 我经常这样
☐ 现在已经不太这样了
☐ 现在肯定是不太多了
☐ 根本没有

5、我的心中充满烦恼：
☐ 偶然如此
☐ 时时，但并不轻松
☐ 时常如此
☐ 大多数时间

6、我感到愉快：
☐ 大多数时间
☐ 有时
☐ 并不经常
☐ 根本没有

7、我能够安闲而轻松地坐着：
☐ 肯定
☐ 经常
☐ 并不经常
☐ 根本没有

8、我对自己的仪容失去兴趣：
☐ 我仍然像以往一样关心
☐ 我可能不是非常关心
☐ 并不像我应该做的那样关心我
☐ 肯定

9、我有点坐立不安，好像感到非要活动不可：
☐ 根本没有
☐ 并不很少
☐ 是不少
☐ 却是非常多

10、我对一切都是乐观地向前看：
☐ 差不多是这样做
☐ 并不完全是这样做的
☐ 很少这样做
☐ 几乎从不这样做

11、我突然发现有恐慌感：
☐ 根本没有
☐ 并非经常
☐ 非常肯定，十分严重
☐ 确实很经常

12、我好像感到情绪在渐渐低落：
☐ 根本没有
☐ 有时
☐ 很经常
☐ 几乎所有时间

13、我感到有点害怕，好像某个内脏器官发生变化了：
☐ 根本没有
☐ 有时
☐ 很经常
☐ 非常经常

14、我能欣赏一本好书或一则好的广播或电视节目：
☐ 常常如此
☐ 有时
☐ 并非经常
☐ 很
