# Supplementary material for: Correlation Between Chronic Pain Acceptance and Clinical Variables in Ankylosing Spondylitis and Its Prediction Role for Biologics Treatment
Source: Front Med (Lausanne). 2020 Jan 31;7:17. doi: 10.3389/fmed.2020.00017 (PMC7005047; doi:10.3389/fmed.2020.00017)
Supplement: Supplementary file 5 [file Data_Sheet_5.PDF]

以下问题是关于您的疼痛与您平时活动的关系,请根据您的实际情况选择你认为最合适的答案,在数字上打勾。

	强烈反对	反对	同意	完全同意
1、如果让我运动，我害怕会受伤。	**1**	**2**	**3**	**4**
2、如果我尝试克服疼痛,疼痛会加剧。	**1**	**2**	**3**	**4**
3、我的身体告诉我，我的处境很危险。	**1**	**2**	**3**	**4**
4、如果我运动，疼痛很可能会缓解。	**1**	**2**	**3**	**4**
5、我觉得人们对我的健康状况不够关心。	**1**	**2**	**3**	**4**
6、意外受伤会使我在今后的日子里一直处于危险之中。	**1**	**2**	**3**	**4**
7、疼痛意味着我的身体受到了伤害。	**1**	**2**	**3**	**4**
8、可以加剧疼痛是事情并不意味着它们很危险。	**1**	**2**	**3**	**4**
9、我常常害怕我会意外受伤。	**1**	**2**	**3**	**4**
10、为了防止疼痛加剧，保持谨慎、不做多余的动作是最安全的。	**1**	**2**	**3**	**4**
11、如果我的体内没有一些潜在的危险因素，我不会感到如此疼痛。	**1**	**2**	**3**	**4**
12、尽管我感到很痛,但如果我积极运动疼痛会缓解。	**1**	**2**	**3**	**4**

	强烈反对	反对	同意	完全同意
13、疼痛时我会停止运动以防止受伤。	**1**	**2**	**3**	**4**
14、我觉得积极运动并不安全。	**1**	**2**	**3**	**4**
15、我很容易受伤，所以无法像常人一样做事。	**1**	**2**	**3**	**4**
16、尽管有些事给我带来了许多疼痛，但我并不认为它们很危险。	**1**	**2**	**3**	**4**
17、我觉得人在疼痛时不应该去运动。	**1**	**2**	**3**	**4**
