# Supplementary material for: Correlation Between Chronic Pain Acceptance and Clinical Variables in Ankylosing Spondylitis and Its Prediction Role for Biologics Treatment
Source: Front Med (Lausanne). 2020 Jan 31;7:17. doi: 10.3389/fmed.2020.00017 (PMC7005047; doi:10.3389/fmed.2020.00017)
Supplement: Supplementary file 6 [file Data_Sheet_6.PDF]

Tampa Scale for Kinesiophobia

With the following questions, we would like to examine how you see your pain. Indicate your degree of agreement with the following statements. Please mark the according number after each statement.

1 = strongly disagree

2 = disagree

3 = agree

4 = strongly agree

1. I'm afraid that I might injury myself if I exercise	1	2	3	4
2. If I were to try to overcome it, my pain would increase	1	2	3	4
3. My body is telling me I have something dangerously wrong	1	2	3	4
4. My pain would probably be relieved if I were to exercise	1	2	3	4
5. People aren't taking my medical condition seriously enough	1	2	3	4
6. My accident has put my body at risk for the rest of my life	1	2	3	4
7. Pain always means I have injured my body	1	2	3	4
8. Just because something aggravates my pain does not mean it is dangerous	1	2	3	4
9. I am afraid that I might injure myself accidentally	1	2	3	4
10. Simply being careful that I do not make any unnecessary movements is the safest thing I can do to prevent my pain from worsening	1	2	3	4
11. I wouldn't have this much pain if there weren't something potentially dangerous going on in my body	1	2	3	4
12. Although my condition is painful, I would be better off if I were physically active	1	2	3	4
13. Pain lets me know when to stop exercising so that I don't injure myself	1	2	3	4
14. It's really not safe for a person with a condition like mine to be physically active	1	2	3	4
15. I can't do all the things normal people do because it's too easy for me to get injured	1	2	3	4
16. Even though something is causing me a lot of pain, I don't think it's actually dangerous	1	2	3	4
17. No one should have to exercise when he/she is in pain	1	2	3	4
